# Supplementary material for: Phylo-Plex: a phylogenetically informed, low-cost amplicon sequencing platform for deployable high-resolution genomic epidemiology
Source: Nat Commun. 2026 Jul 9;17:5839. doi: 10.1038/s41467-026-75002-y (PMC13350907; doi:10.1038/s41467-026-75002-y)
Supplement: Supplementary file 2 — Description of Additional Supplementary Files [file 41467_2026_75002_MOESM2_ESM.pdf]

Description of Additional Supplementary Files

File Name: Supplementary_Data_1.xlsx
Metadata and list of genomes used for designing the TP-Phylo-Plex scheme.

File Name: Supplementary_Data_2.xlsx
Primers designed for TP-Phylo-Plex scheme.

File Name: Supplementary_Data_3.xlsx
Description of sequences and metadata for validation samples from South Africa.

File Name: Supplementary_Data_4.xlsx
Description of sequences and metadata for field work samples from Zimbabwe.

File Name: Supplementary_Data_5.xlsx
Cost breakdown of applying the Phylo-Plex method to MLST.

File Name: Supplementary_Data_6.csv
Genomic regions inferred for eQicient reconstruction of *Neisseria gonorrhoeae* transmission clusters.
