# Supplementary material for: Phylo-Plex: a phylogenetically informed, low-cost amplicon sequencing platform for deployable high-resolution genomic epidemiology
Source: Nat Commun. 2026 Jul 9;17:5839. doi: 10.1038/s41467-026-75002-y (PMC13350907; doi:10.1038/s41467-026-75002-y)
Supplement: Supplementary file 9 — Reporting Summary [file 41467_2026_75002_MOESM9_ESM.pdf]

Corresponding author(s): Mathew Beale

Last updated by author(s): 06/05/2026

Reporting Summary

Nature Portfolio wishes to improve the reproducibility of the work that we publish. This form provides structure for consistency and transparency in reporting. For further information on Nature Portfolio policies, see our Editorial Policies and the Editorial Policy Checklist.

Statistics

For all statistical analyses, confirm that the following items are present in the figure legend, table legend, main text, or Methods section.

n/a	Confirmed	
☐	☒	The exact sample size (*n*) for each experimental group/condition, given as a discrete number and unit of measurement
☒	☐	A statement on whether measurements were taken from distinct samples or whether the same sample was measured repeatedly
☐	☒	The statistical test(s) used AND whether they are one- or two-sided<br>*Only common tests should be described solely by name; describe more complex techniques in the Methods section.*
☒	☐	A description of all covariates tested
☒	☐	A description of any assumptions or corrections, such as tests of normality and adjustment for multiple comparisons
☐	☒	A full description of the statistical parameters including central tendency (e.g. means) or other basic estimates (e.g. regression coefficient) AND variation (e.g. standard deviation) or associated estimates of uncertainty (e.g. confidence intervals)
☐	☒	For null hypothesis testing, the test statistic (e.g. *F*, *t*, *r*) with confidence intervals, effect sizes, degrees of freedom and *P* value noted<br>*Give P values as exact values whenever suitable.*
☒	☐	For Bayesian analysis, information on the choice of priors and Markov chain Monte Carlo settings
☒	☐	For hierarchical and complex designs, identification of the appropriate level for tests and full reporting of outcomes
☒	☐	Estimates of effect sizes (e.g. Cohen's *d*, Pearson's *r*), indicating how they were calculated

Our web collection on statistics for biologists contains articles on many of the points above.

Software and code

Policy information about availability of computer code

Data collection

No software was used for data collection.

Data analysis

Illumina Sequence reads were taxonomically classified using Kraken2 v2.0.8 with the full bacterial and human database (2019-03-29). Reads were trimmed using Trimmomatic v0.39 and downsampled using seqtk v1.0. Simulated reads were generated from published assemblies using Fastaq v3.17.0. The SS14 reference genome was masked for known regions of uncertainty/recombination using bedtools v2.29 maskfasta. Sequencing reads were mapped to the reference genome using BWA mem v0.7.17, followed by indel realignment using GATK v3.7, deduplication with Picard MarkDuplicates v1.126, and variant calling with samtools v1.6 and bedtools v1.6 to generate a pseudosequence and multiple sequence alignment. We repeated masking of the known regions using remove_block_from_aln.py (available at https://github.com/sanger-pathogens/remove_blocks_from_aln/).

For phylogenetic analysis, we converted whole genome length multiple sequence alignments to SNP-only alignments using snp-sites v2.5.1 (available at https://github.com/sanger-pathogens/remove_blocks_from_aln/, commit a274ec4e47528a4f1ad7d9445d7a416fe8994397). Maximum likelihood phylogenies were calculated using IQ-Tree v1.6.12. We used Gubbins v2.4.1 to identify and mask putative regions of recombination. For phylogenetic clustering, we used pyjar v0.1.0 (available at https://github.com/simonrharris/pyjar/, commit 87e93a1c909a6024990a56d3a88dc82627315b27), and rPinecone v0.1.0 (available at https://github.com/alexwailan/rpinecone, commit 36114f5795701340660c141e8269dbeddf53e72a).

MinION data from initial validate runs was basecalled and demultiplexed using Guppy v6.4.2 with the High Accuracy model, and coverage was analysed using samtools v1.6 depth with a custom python script available at https://github.com/matbeale/TP-Phylo-Plex_paper_2025/blob/main/scripts/Coverage_over_defined_regions_v0.1.py. Variants were called using Clair3 v1.0.1. MinION variant calls were merged with those from Illumina data for the same samples using bcftools v1.19.

We subsequently built a NextFlow pipeline for automated data handling available at https://github.com/sanger-pathogens/ONTAP. This used Dorado v0.5.1 as the basecaller using the "dna_r10.4.1_e8.2_400bps_hac@v4.3.0" model. Primers were trimmed from amplicons using Cutadapt v4.7, and low quality base regions were masked using seqtk v1.4. Sequencing reads were mapped to the reference genome using minimap2 v2.26. Variant calling was performed using Clair3 v1.0.9 in haploid mode, using the "r1041_e82_400bps_hac_v430" model. Bcftools v1.20 was used to merge variant calls. A custom script available at https://github.com/sanger-pathogens/ONTAP/gvcf_to_fasta.py was used to create a consensus fasta sequence for each sample and amplicon region. Additional quality control was conducted using PycoQC v2.5.2, FastQC v0.12.1, samtools v1.6, bedtools v2.31.1 and MultiQC v1.22.2. Additional scripts available at https://github.com/sanger-pathogens/ONTAP/tree/main/bin were used for generating additional QC outputs. Analysis of ZImbabwe data conducted in the field was performed using https://github.com/sanger-pathogens/ONTAP v1.0.0, and interactive interpretation was performed using a Shiny App available at https://github.com/sanger-pathogens/ONTAP/blob/main/AmpliSeq_QC_Frontend.R.

Phylogenies and tanglegrams were plotted using ggtree v3.12.0, and all other plots were produced using ggplot2 v3.5.1 in R v4.4.2. Networks were plotted using ggnetwork v0.5.13and iGraph v2.0.3, and all statistics were calculated in R.

Multiple sequence alignments from the base dataset were converted into multi-VCF format using snp-sites v2.5.1, and imported into R using vcfR v1.15.0. FST was calculated using the `wc` command from the hierfstat v0.5.11 package in R. Networks were inferred using the network v1.18.2 package, and network components were extracted using iGraph v2.0.3.

Multiplex PCR primers were designed using PrimalScheme v1.3.2, using the option to input multiple different sequence alignments (each representing a different target region). We used the primerTree v1.0.6 package API to conduct primer BLAST searches against the NCBI database. We validated primer hits using in_silico_pcr.py (available at https://github.com/sangerpathogens/sh16_scripts/blob/master/legacy/in_silico_pcr.py).

To identify conserved regions suitable for macrolide resistance testing, we performed Compact Bit-Sliced Signature Index using the index provided with Blackwell 2021 for ~661,000 uniformly assembled bacterial genomes against 71bp sliding windows taken from the appropriate T. pallidum reference genome (NC_021508.1) ribosomal 23S region.For selected regions, primers were designed using PrimalScheme v1.3.2 as above.

To evaluate the scheme, we initially simulated amplicons from Illumina-derived whole genomes. Based on the target regions, we extracted SNPs and constructed multiple sequence alignments using the vcfR v1.15.0, ape v5.8 and seqinr v4.2.36 packages, from which we inferred phylogenies using IQ-Tree v1.6.12. We compared the whole genome phylogenies to the inferred amplicon phylogenies for phylogenetic consistency using tanglegrams produced using ggtree v3.12.0, distance matrix comparison using Mantel tests in vegan v2.6.6.1, as well as correlating whole genome derived sublineages with amplicon derived phylogenies using the treeConcordance function in Treespace v1.1.4.3.

To evaluate the ability of Phylo-Plex to detect novel variation, we used a custom python script (randomly_mutate_genome.py, available at https://github.com/matbeale/TP-Phylo-Plex_paper_2025/blob/main/scripts/randomly_mutate_genome.py).

The code used for identifying discriminatory sites, for identifying positional network clusters, iterative region selection, and all plots and statistics used are available in a interactive Rnotebook at https://github.com/matbeale/TP-Phylo-Plex_paper_2025/blob/main/Treponema_Phylo-Seq_Discrimintory_amplicon_analysis_04-2025.Rmd.

For manuscripts utilizing custom algorithms or software that are central to the research but not yet described in published literature, software must be made available to editors and reviewers. We strongly encourage code deposition in a community repository (e.g. GitHub). See the Nature Portfolio guidelines for submitting code & software for further information.

Data

Policy information about availability of data

All manuscripts must include a data availability statement. This statement should provide the following information, where applicable:

- Accession codes, unique identifiers, or web links for publicly available datasets
- A description of any restrictions on data availability
- For clinical datasets or third party data, please ensure that the statement adheres to our policy

European Nucleotide Archive (ENA; EMBL-EBI) Accessions for Illumina sequencing reads from the initial dataset used for primer design are listed in Supplementary Table 1. Accessions for the Illumina reads from South African syphilis are listed in Supplementary Table 3 and are available at the ENA under project accession PRJEB60271. Oxford Nanopore sequencing reads are available from the ENA under project accession PRJEB85457, and are listed in Supplementary Table 3 (for South Africa) and Supplementary Table 4 (for Zimbabwe).

All files used in the Rnotebook analysis (including raw and intermediate files) are available at https://github.com/matbeale/TP-Phylo-Plex_paper_2025 and at https://doi.org/10.5281/zenodo.14894111.

Research involving human participants, their data, or biological material

Policy information about studies with human participants or human data. See also policy information about sex, gender (identity/presentation), and sexual orientation and race, ethnicity and racism.

Reporting on sex and gender	This data was not available, and is not relevant to the analyses presented in the manuscript.

Reporting on race, ethnicity, or other socially relevant groupings	This data was not available, and is not relevant to the analyses presented in the manuscript.
Population characteristics	Patients were recruited through polyclinics in Harare, Zimbabwe. Patients were all over the age of 16 (the local age of majority) and gave written informed consent for participation in the study. For the overall study, 50% of individuals recruited self-described as male and 50% were female.
Recruitment	Patients with symptomatic genital ulcers were recruited into an observational study in Zimbabwe as part of a Multi-Country Aetiology of Genital Ulcer Survey (MAGUS). Patients were recruited through polyclinics in Harare, Zimbabwe. For the overall study, 50% of individuals recruited self-described as male and 50% were female. There were no specific biases expected, since we focussed on symptomatic individuals. Moreover, such biases would not be relevant to the goals or outcomes of this study, which used those samples largely for the purposes of evaluating a method.
Ethics oversight	Clinical samples from South Africa were collected as part of routine public health surveillance, and ethical approval for genome sequencing was granted by the University of the Witwatersrand Human Research Ethics Committee (Medical) (Ethics clearance certificate no. M230157). Clinical swabs from Zimbabwe were collected as part of the Multi-Country Aetiology of Genital Ulcer Study (MAGUS), and ethical approvals were granted for patient recruitment, diagnostics and genomics from the Medical Research Council of Zimbabwe (MRCZ/A/2878), the Biomedical Research and Training Institute Institutional Review Board (Ap/175/2022) and the London School of Hygiene and Tropical Medicine Ethics Committee (26731). Samples were collected with informed consent for molecular diagnostics, genomics and metagenomics.

Note that full information on the approval of the study protocol must also be provided in the manuscript.

Field-specific reporting

Please select the one below that is the best fit for your research. If you are not sure, read the appropriate sections before making your selection.

☒ Life sciences ☐ Behavioural & social sciences ☐ Ecological, evolutionary & environmental sciences

For a reference copy of the document with all sections, see nature.com/documents/nr-reporting-summary-flat.pdf

Life sciences study design

All studies must disclose on these points even when the disclosure is negative.

Sample size	The dataset used for design was chosen to be representative of the known genomic diversity across Treponema pallidum at the time of commencing the work (Summer 2022). Samples from South Africa were selected from an ongoing study on genomic diversity in that country. We selected 72/101 samples with the lowest T. pallidum qPCR Ct for validation - this represented three flowcells worth of samples (24 samples per flowcell). Samples from Zimbabwe were collected as part of an ongoing surveillance study - we collected 108 genital ulcer samples from different patients, and screened all samples for T. pallidum, identifying 14 samples with T. pallidum. All 14 samples were sequenced, and we selected two samples to be sequenced in various combinations of technical replicate.
Data exclusions	After initial evaluation of our PCR amplicon sequencing scheme, we identified 15/74 amplicons (Phy-Cons) which consistently yielded reduced coverage in multiplex PCR (but performed well as single-plex PCR assays). For the purposes of the scheme development, we removed these amplicons and retrospectively evaluated the impact on discriminatory power for sublineages.
Replication	In silico analyses were performed using the original T. pallidum design to confirm phylogenetic placement was as expected by extracting amplicon regions from the dataset used for design. We confirmed the approach to identifying candidate regions works by replicating in a different pathogen, Neisseria gonorrhoeae, which has different population genomic characteristics (much greater diversity), in addition to in silico analysis of the impact of increasing diversity on region clustering and needed amplicon length.<br><br>For Laboratory testing, we evaluated the original laboratory testing using dilution series and replicates of a small panel of reference samples with known genomes. For the South African validation set, we directly compared amplicon-derived sequences to those from independently performed Illumina whole genome sequencing, comparing sequences for the amplicon regions and investigating a small number of discrepancies (described in the Results). For two samples from Zimbabwe, we performed independent DNA extraction and sequencing as technical replicates; for one of these samples, we also sequenced the same extract twice as a further technical replicate.These replicates had identical amplicon sequences.
Randomization	Not relevant - we were designing, developing and evaluating a method, not performing an experimental study or trial.
Blinding	Amplicon sequencing was performed blind to the variants that were present in the sample, and amplicon variant calls were only linked to comparator Illumina WGS variant calls for comparison.

Reporting for specific materials, systems and methods

We require information from authors about some types of materials, experimental systems and methods used in many studies. Here, indicate whether each material, system or method listed is relevant to your study. If you are not sure if a list item applies to your research, read the appropriate section before selecting a response.

Materials & experimental systems

n/a	Involved in the study
☒	☐ Antibodies
☒	☐ Eukaryotic cell lines
☒	☐ Palaeontology and archaeology
☒	☐ Animals and other organisms
☒	☐ Clinical data
☒	☐ Dual use research of concern
☒	☐ Plants

Methods

n/a	Involved in the study
☒	☐ ChIP-seq
☒	☐ Flow cytometry
☒	☐ MRI-based neuroimaging

Plants

Seed stocks	*Report on the source of all seed stocks or other plant material used. If applicable, state the seed stock centre and catalogue number. If plant specimens were collected from the field, describe the collection location, date and sampling procedures.*
Novel plant genotypes	*Describe the methods by which all novel plant genotypes were produced. This includes those generated by transgenic approaches, gene editing, chemical/radiation-based mutagenesis and hybridization. For transgenic lines, describe the transformation method, the number of independent lines analyzed and the generation upon which experiments were performed. For gene-edited lines, describe the editor used, the endogenous sequence targeted for editing, the targeting guide RNA sequence (if applicable) and how the editor was applied.*
Authentication	*Describe any authentication procedures for each seed stock used or novel genotype generated. Describe any experiments used to assess the effect of a mutation and, where applicable, how potential secondary effects (e.g. second site T-DNA insertions, mosiacism, off-target gene editing) were examined.*
