# Supplementary material for: Phylo-Plex: a phylogenetically informed, low-cost amplicon sequencing platform for deployable high-resolution genomic epidemiology
Source: Nat Commun. 2026 Jul 9;17:5839. doi: 10.1038/s41467-026-75002-y (PMC13350907; doi:10.1038/s41467-026-75002-y)
Supplement: Supplementary file 10 — Transparent Peer Review file [file 41467_2026_75002_MOESM10_ESM.pdf]

nature portfolio

Peer Review File

Phylo-Plex: A phylogenetically informed, low-cost amplicon sequencing platform for deployable high-resolution genomic epidemiology

Corresponding Author: Dr Mathew Beale

This file contains all reviewer reports in order by version, followed by all author rebuttals in order by version.

Version 0:

Reviewer comments:

Reviewer #2

(Remarks to the Author)
In the presented work, Beale et al. designed a systematic approach for selection of the smallest number of variable regions in a bacterial genome that would allow high resolution surveillance and classification of clinical isolates. The resulting typing method, denoted Phylo-Plex, utilizes PCR amplification of bacterial DNA directly from clinical specimen, followed by nanopore sequencing. The authors offer it as an alternative to genomic pathogen surveillance that uses whole genome sequencing due to the high resolution and low cost. They proposed it to be ideal for use in low-income settings.
Beale et al. provided proof-of-concept using Treponema pallidum subspecies as models. The authors designed 59 amplicons (denoted Phylo-cons) that classified Treponema pallidum isolates into 40 sub-lineages matching available whole genome sequencing (WGS) data. Of those 40, 17 sub-lineages were of Treponema pallidum subsp. pallidum (TPA), which causes syphilis. The authors then tested the resulting approach on syphilis-positive samples from South Africa (N=72) and Zimbabwe (N=14).

General comments: The manuscript is well written, easy to follow, and accompanied by clear and high-quality figures.

Noteworthy results & Significance: The authors present a useful and logical approach to select the minimal number of genome regions that allow precise typing of bacterial isolates on par with WGS. The code is available for researchers to use on their pathogens of choice, which also adds to transparency and reproducibility of the results. The results are solid and of value to the research community.

Comparison to the established literature: Direct comparisons with the commonly implemented methods are needed. For example, the cited MLST scheme has been used to type approximately 1,600 Treponema pallidum isolates from around the world, showing over 110 different allelic profiles (data are publicly available through the PubMLST database). The current manuscript would benefit from more transparent comparison of the new method with this scheme. Additional evidence is, therefore, needed.

The methodology is sound and meets the expected standards. Enough detail is provided for the work to be reproduced.

Specific comments are listed below.

Authors compare the new Phylo-Plex scheme to the existing MLST scheme at multiple places of the manuscript. They conclude that the Phylo-Plex offers more resolution and is less costly. Data supporting these claims should be included.
- It is unclear what the output of the Phylo-Plex typing is. Is it an assignment of an isolate to a TPA sub-lineage? If this is the case, would it not provide lower resolution than MLST (17 TPA sub-lineages vs. over 110 different allelic profiles using MLST)? Clarification is needed.
- Supplementary Figure 12: It would be helpful to construct the tree using the MLST scheme for comparison.
- A direct comparison of cited methods using the same set of clinical sample is warranted to highlight the better resolution of the Phylo-Plex when compared to the traditional typing methods.

- The lower price is highlighted by comparing Phylo-Plex via nanopore sequencing versus MLST via Sanger sequencing. These two sequencing methods have very different starting costs, which are unrelated to the typing schemes. What would be the cost if the MLST was performed using nanopore sequencing? If MLST uses 4 (3 discriminatory + 23S rRNA) instead of 59 amplicons, the overall price is expected to be lower.
- The WGS price is listed for Illumina sequencing, which is again costlier than nanopore. What is the estimated price of sequencing TPA genome following DNA capture using nanopore?

The authors mention several times that their new approach can be applied to any pathogen. Treponema pallidum has very small and conserved genome with minimal differences, even between subspecies that cause distinct diseases. Authors state that more variable genomes would be even more suitable for Phylo-Plex development. However, the opposite is also possible with more Phylo-cons potentially needed to obtain the desired resolution, increasing price and data processing needs. It would be helpful to support this claim using a different pathogen. For example, estimate how many Phylo-cons would be needed to develop Phylo-Plex in a bacterium with more "standard" genome.

Finally, how are the results of Phylo-Plex evaluated in low-income settings?

(Remarks on code availability)

Reviewer #3

(Remarks to the Author)
The authors developed a strategy to design a typing method based on targeted sequencing of phylogenetically informative regions across a set of genomes. Their method is focused on identifying target regions that are suitable for amplification in single-tube multiplex PCR reactions prior to sequencing. They use their strategy to design a typing scheme for T. pallidum, but their method could indeed be used for other organisms. Overall, I find the study scientifically sound and interesting. The authors follow a rational and phylogenetically-driven approach to build their typing scheme which, in my opinion, is one of the best ways a sub-genomic typing method could be developed (if not the best). I think the methodology of the authors overall makes sense, and their results overall back their claims. Therefore, I would recommend this work for publication after some corrections have been made to improve the manuscript, clarify some parts, and better discuss some points, in which I believe the authors may have not been completely fair. Importantly, I believe that the authors have missed relevant previous studies which should be discussed in their manuscript.

I hope that the authors will find our comments useful, all of which are constructively provided with the intention of improving the manuscript.

Sincerely,
Dr. Galo A. Goig and Gian Schüpbach

Major Comments:

The authors have missed relevant literature and should put their work in the context of previous studies (see Seabolt et al. Tau-typing: a Nextflow pipeline for finding the best phylogenetic markers in the genome for molecular typing of microbial species, Bioinformatics 2023).

The authors say they use a "globally representative dataset of T. Pallidum genomes". The input dataset used for this kind of work is pivotal in the design of the scheme, however the authors do not provide references, nor explain, why the reader should believe that this dataset is globally representative. Also, representative of what exactly? Of the worldwide circulating genetic diversity? As for many other pathogens, T. Pallidum disproportionately affects LMICs, yet more than two thirds of the genomes analyzed in this study come from three high-income countries (Canada, The UK and USA). I understand that there may not be much WGS data available from LMICS, and I find acceptable that the authors just use what they have. But, is this dataset really representative of the global genetic diversity of T. Pallidum, given that for some high-incidence settings there are available no more than a couple of genomes? The authors should discuss the limitations of their typing scheme in this regard.

I think the introduction of this paper lacks a couple of introductory sentences to i) the global genetic diversity of T. Pallidum. ii) The current typing methods available and more commonly used for T. Pallidum. This background is necessary to better evaluate the advantages of TP-Phylo-plex.

Along the same lines, in the discussion section, the authors highlight the advantages of their methodology as opposed to other alternatives. I believe they are not fair in this comparison, by presenting overly pessimistic alternatives as compared to Phylo-Plex. For example, the authors say that MLST, "requires" sanger sequencing to then come up with an estimated cost of 28-112 pounds per sample, as compared to 12 of PhyloPlex. I do not see why MLST amplicons could not be sequenced with other technologies. As a matter of fact, the platform used by the authors (ONT) could be used for MLST schemes for the exact same reasons and with the same advantages and disadvantages than PhyloPlex. I believe the discussion should be rather focused on what value PhyloPlex adds as compared to alternatives such as MLST, but I doubt the decisive factor will be cost. Similarly, when the authors claim about Phylo-Plex: "because it can use multiple sequencing technologies, it can be scaled…" would apply to all other sequencing-based methods as well.

I do not understand the rationale to exclude the 15 amplicons from the initial Phylo-Plex scheme. In Supplementary Figure 9, it is very clear that the amplification efficiency for some amplicons is poor, and therefore the authors exclude them from the scheme. This is very clear for amplicons 1, 16, 37, 49, 67, 71, and maybe 38. However, some other amplicons do not seem problematic to me at all, and still the authors decided to exclude them in principle because they are part of the 5% that performed worst if I understood correctly. It does not seem to be a reasonable criterion to exclude regions, since even if all amplicons perform perfectly well, there will be always a 5% performing worst. For example, why were regions 20, 50 or 61 excluded? This is particularly relevant, given that exclusion of original amplicons led the authors to a rather disappointing discrimination power of around 75% of the original target. I also find confusing that the authors say in the text that, after exclusion, they discriminate 31/40 sublineages, but in Supplementary Figure 4 only 8, instead of 9, are pointed as removed.

I think that readers that do not realize that the strength of this work is its approach to design typing schemes, and not the scheme itself (which performs poorly as it is), may find the results disappointing: at the end of the day all this work to “only” discriminate 75% of the initial target sublineages. I would suggest to make this point very clear in the manuscript, and also discuss that a multiplex reaction always needs optimization (different concentrations of primers, inclusion of other amplicons from the initial pool of >800 amplicons, etc), something that was not carried out in this work. I only found such a comment in the legend of supplementary figure 4.

Together with this low “sensitivity” of the scheme in its actual form, the authors report 3 samples with discordant calls between Illumina and ONT. This means 5% of the samples tested. The authors say they implemented improvements in the pipeline to account for problematic samples with low coverage in the future. I am not sure if these improvements are the ones described in the Methods section but most importantly, instead of limiting to say that these improvements will deal with these problematic samples in the future, why did not the authors reanalyze the data and demonstrate that these improvements are able to deal with these discrepancies in this present study?

“The median genome count for each window was 129 (reflecting the number of T. pallidum genomes in the dataset), and regions of the T. pallidum 23S operon identified as matching ≥5% above the median (135.5) were flagged as potentially cross-species matches and masked to avoid use in primer design” If the number of TP genomes in the dataset is 129, why is 129 not the threshold, instead of 5% of the median? That would mean that around 6 species which are not TP may amplify, and precisely given how conserved this gene is, and its implications in drug resistance, I do not think this is an appropriate criterion.

Minor comments:

The authors say that the TP-Phylo-Plex scheme was straightforward for TP given the low genetic diversity and little recombination. They could comment on how feasible would be to design schemes for pathogens with higher diversity.

“Notably, those sublineages occurred in clonal lineages such as TPA SS14-Lineage which had the smallest number of available discriminatory sites even at the WGS level, and were thus most akected.” I find this statement misleading. In the tree of Supplementary Figure seems that most of the unclassified samples would have enough diversity and therefore that the reason why discrimination was not possible was rather the exclusion of the amplicons that contained their defining SNPs, no the fact that discrimination is impossible between highly clonal strains.

“Then, regardless of the nature of any individual SNP or gene, all discriminatory SNPs are positionally clustered…” What do the authors mean by “the nature of the SNPs” exactly? This is unclear, and if they mean “regardless of the genomic region in which these SNPs are”, then it would not be accurate since their analysis explicitly excludes hypervariable, recombining and repetitive regions.

The authors built a ML phylogeny with IQ-TREE, but it is not specified the model of evolution, bootstrap replicates, etc…

“To identify highly conserved Treponema specific regions in the 23S gene, which occurs universally at high homology across bacteria” please substitute homology by another term such as similarity. A trait is either homologous or non-homologous, it cannot be more or less homologous.

I suggest to improve figure 8 by overlaying the points to boxplots. I also suggest to include a plot where the correlation (or lack thereof) between qPCR Ct and average sample depth is shown.
Supplementary 8, together with Suppl 9, show no correlation between Ct and depth, this means that Supplementary Figure 9 could be simplified and would be more informative if eliminating the X axis (Ct), and simply plotting boxplots and datapoints of depth for each amplicon.

In the bibliography, species names are not in italics.

(Remarks on code availability)

Reviewer #4

(Remarks to the Author)
I co-reviewed this manuscript with one of the reviewers who provided the listed reports. This is part of the Nature

Communications initiative to facilitate training in peer review and to provide appropriate recognition for Early Career Researchers who co-review manuscripts.

(Remarks on code availability)

Reviewer #5

(Remarks to the Author)
The manuscript entitled “Phylo-Plex: A phylogenetically informed, low-cost amplicon sequencing platform for deployable high-resolution genomic epidemiology” describes a framework for the design of an amplicon panel for targeted culture-independent sequencing. This framework is focused on the acquisition of genomic information though the identification of ancestral SNPs, that will be useful for the genomic epidemiology of pathogens of public and global health concerns. The authors applied this framework to develop a multiplex PCR panel capable of distinguishing between sub lineages of T. pallidum. This resulted in a final pool of 59 primer set. The authors further validated the panel in a low-resource laboratory setting, demonstrating its ability to recover amplicon profiles from clinical samples with Ct values ≤32, consistent with their initial validation.

The manuscript reads well and the framework will be a very useful tool for the design of amplicon panels, targeting hard-to-culture pathogens (with good genomic representation in public genomic repositories). While I recommend publication, there are a few (mostly minor) comments to be addressed.

Major comments
• Dropping the number of amplicons from 72 resulted in the loss of discrimination from 9 of the initial targeted 40 sub lineages. As the authors mentioned that singleplex PCR all performed ok, was the generation of multiple primer pools to reduce primer cross interactions considered/investigated to mitigate this loss in resolution?

• Targeted culture-independent sequencing can also be achieved through other methods via ONT sequencing. One such method is ONT Adaptive Sampling, which requires no prior primer amplification and uses the same hardware for sequencing. While the role of amplicon sequencing for public health purposes is not in doubt, deeper discussion points between the various culture independent sequencing methods would provide valuable context and enhance the depth of the discussion.

Other comments
• Page 7: It was mentioned in-text that there were 72 amplicons and 1x 23S amplicon. However, supplementary table 2 had 72 amplicons and 2 x 23S amplicons listed. The second 23S amplicon did not make it into the final set and was not mentioned in-text. Why was this primer dropped?

• Page 10: This sentence needs clarification: “Of the two discrepant sites, the first (NC_021508.1 position 7002) affected 1/59 samples and was due to a deletion detected in the Illumina sequence (7002T) compared to the reference and MinION sequences (7002TG).” Do the authors mean that a T deletion at position 7002 (7002delT) was observed only in the Illumina reads and not in the ONT reads (prior to closer inspection)? If so, I recommend referring to it as 7002delT and omitting the “7002TG” notation, since positions 7002 and 7003 in NC_021508.1 are T and G, respectively.

• Page 13: During the implementation study, two samples had one technical replicate each and one sample had three replicates. Two samples (mz454 & mz446) had CT values that are different within their replicates (from Supplementary Table 4), are these values listed in supplementary table correct or are they biological replicates? In addition, why did three samples have replicates but not the others?

• Page 13: From cross checking of the data in Supplementary Table S4, did the authors mean “5 samples, 3 of which are replicates of a single sample” instead of “(3 samples (5 replicates)” In addition, mz454a with a CT of 25 (PolA) also resulted in a failed run, which should be mentioned in text.

• Page 13: Authors mentioned that 12 TP-Phylo-Plex profiles were generated without going into details on the sublineages called. Can the authors report on the sub lineage calls of each of the profile (either in text or in supplementary). Also, one of the samples that was successful in sequencing also had a technical replicate- can the authors comment if the technical replicates resulted in the same sublineage call?

• Page 13: The estimated cost per sample does not include DNA extraction. It is understandable why this was not included as the focus of the implementation was on primer scheme. I recommend that the following be added to the sentence for completeness “…(Table 1), in addition to existing costs for DNA extraction and quality control.”

• Page 21: DNA extraction method not listed within laboratory workflows. Please include details on extraction methodology used in this study.

• Page 22: Change the catalogue numbers of the Native barcoding library preparation kits to the SQK designations instead of the auxiliary EXP designation.

• Page 23: Please include the model used in Clair3 for the processing of the R9 data.

• Supp. Figure 8: In text, the quality metric mentioned was <50X but the legend for the supplementary figure stated that a blue line was drawn at 25 X. Do provide an updated figure with the line drawn at 50X to be in-line with what was mentioned in-text.

• Supp. Figure 12: Image resolution of the minimum spanning tree in Panel B is lower than Panel A. Please provide a higher resolution figure for Panel B

(Remarks on code availability)

Version 1:

Reviewer comments:

Reviewer #2

(Remarks to the Author)
My concerns have been fully addressed in the revision. I do not have any additional comments.

(Remarks on code availability)

Reviewer #3

(Remarks to the Author)
The authors have answered all my questions and addressed all issues. I have no further comments.

Sincerely,
Dr. Galo A. Goig

(Remarks on code availability)

Reviewer #4

(Remarks to the Author)
I co-reviewed this manuscript with one of the reviewers who provided the listed reports. This is part of the Nature Communications initiative to facilitate training in peer review and to provide appropriate recognition for Early Career Researchers who co-review manuscripts.

(Remarks on code availability)

Reviewer #5

(Remarks to the Author)
I thank the authors for their responses and revisions. The authors have addressed all the comments I raised in the previous review, and have no additional comments.

(Remarks on code availability)

Open Access This Peer Review File is licensed under a Creative Commons Attribution 4.0 International License, which permits use, sharing, adaptation, distribution and reproduction in any medium or format, as long as you give appropriate credit to the original author(s) and the source, provide a link to the Creative Commons license, and indicate if changes were made.
In cases where reviewers are anonymous, credit should be given to 'Anonymous Referee' and the source.
The images or other third party material in this Peer Review File are included in the article's Creative Commons license, unless indicated otherwise in a credit line to the material. If material is not included in the article's Creative Commons license and your intended use is not permitted by statutory regulation or exceeds the permitted use, you will need to obtain permission directly from the copyright holder.
To view a copy of this license, visit https://creativecommons.org/licenses/by/4.0/

REVIEWER COMMENTS

Reviewer #2 (Remarks to the Author):

In the presented work, Beale et al. designed a systematic approach for selection of the smallest number of variable regions in a bacterial genome that would allow high resolution surveillance and classification of clinical isolates. The resulting typing method, denoted Phylo-Plex, utilizes PCR amplification of bacterial DNA directly from clinical specimen, followed by nanopore sequencing. The authors offer it as an alternative to genomic pathogen surveillance that uses whole genome sequencing due to the high resolution and low cost. They proposed it to be ideal for use in low-income settings.

Beale et al. provided proof-of-concept using Treponema pallidum subspecies as models. The authors designed 59 amplicons (denoted Phylo-cons) that classified Treponema pallidum isolates into 40 sub-lineages matching available whole genome sequencing (WGS) data. Of those 40, 17 sub-lineages were of Treponema pallidum subsp. pallidum (TPA), which causes syphilis. The authors then tested the resulting approach on syphilis-positive samples from South Africa (N=72) and Zimbabwe (N=14).

General comments: The manuscript is well written, easy to follow, and accompanied by clear and high-quality figures.

Noteworthy results & Significance: The authors present a useful and logical approach to select the minimal number of genome regions that allow precise typing of bacterial isolates on par with WGS. The code is available for researchers to use on their pathogens of choice, which also adds to transparency and reproducibility of the results. The results are solid and of value to the research community.

We appreciate this positive appraisal of our work.

Comparison to the established literature: Direct comparisons with the commonly implemented methods are needed. For example, the cited MLST scheme has been used to type approximately 1,600 Treponema pallidum isolates from around the world, showing over 110 different allelic profiles (data are publicly available through the PubMLST database). The current manuscript would benefit from more transparent comparison of the new method with this scheme. Additional evidence is, therefore, needed.

Many thanks for this question. The real benefits of Phylo-Plex are that it provides an efficient way to design primers for surveillance that accurately reflect the current whole genome phylogeny or a local population structure, and that this is flexible and able to incorporate new diversity, unlike a typing scheme like MLST which, by definition, must remain fixed.

Moreover, given that most *T. pallidum* genomic data are metagenomically derived and frequently contain uncertain genomic positions due to low coverage, this approach enables delineation of informative SNPs whilst accommodating missing data, enabling efficient *in silico* analysis (i.e. classification of genomes), which contrasts with gene-centric approaches which may struggle because individual sites within a locus have insufficient coverage, causing the entire locus to be untypeable.

However, to address your question directly, we used 530 *Treponema* subspecies *pallidum* genomes. Using the Grillova MLST scheme (https://github.com/tseemann/mlst), we classified 373/530 TPA genomes into distinct high confidence MLST ST profiles, including 30 known STs and 13 novel allele combinations not included in the PubMLST database. Next, we mapped both the MLST STs and the Phylo-Plex sublineages to the whole genome phylogeny - this shows that whilst the sublineages we used for our Phylo-Plex scheme are phylogenetically consistent, the MLST STs are not, with arbitrary ST changes within nested phylogenetic clades (see figure below). A good example would be ST2 (in bright green) which occurs in two clearly distinct clades, as well as sporadically as singletons throughout the tree. This is explained by the inclusion of hypervariable genes in the MLST scheme against the general lack of variation seen across the genome.

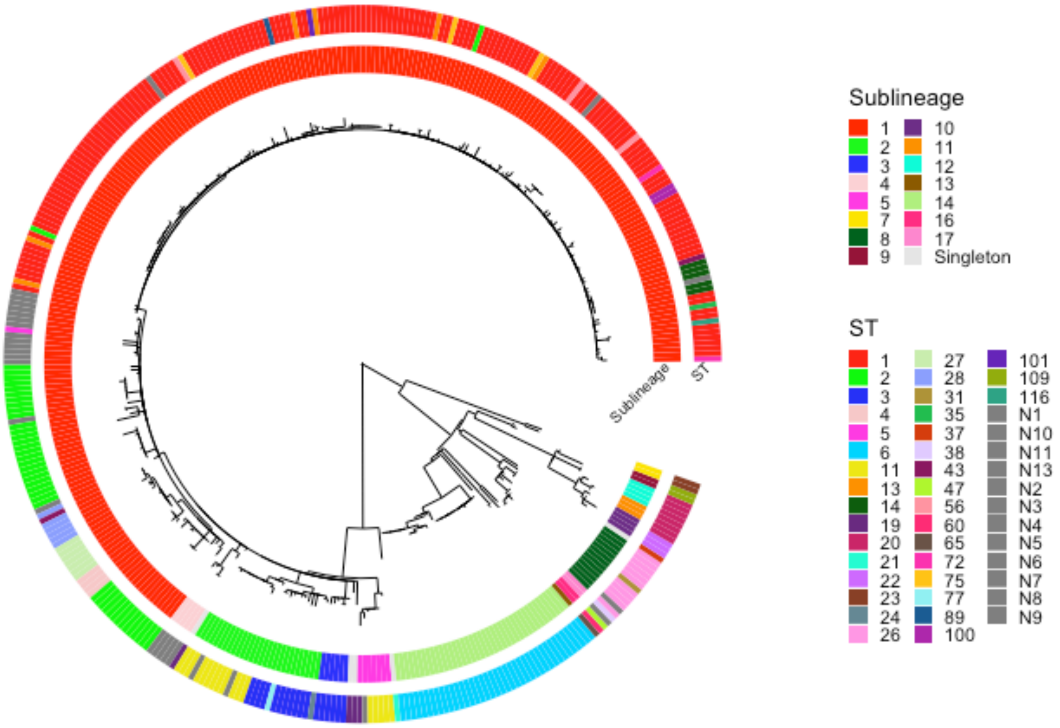

Whilst revising our manuscript in response to these comments, a preprint (https://www.biorxiv.org/content/10.1101/2025.07.10.664125v1) was released describing a new 7-gene MLST scheme for TPA. This scheme uses some of the same hypervariable genes as the previous scheme, and some STs appear genomically very similar to others in the author's WGS phylogeny, but there are no observable homoplasies (the authors did not perform a direct comparison to the original 3-gene scheme).

Given that Phylo-Plex is not intended as a fixed tying-scheme and we are not advocating for it to replace MLST *per se,* we feel that introducing direct comparisons between Phylo-Plex and multiple MLST schemes to the manuscript would substantially distract from the focus of our manuscript, and have therefore not put the above comparison in the paper. However, we recognise that the language used in our original draft implied that these were competitor techniques, leading to the comments here and from other reviewers. We have therefore attempted to strengthen the language around how Phylo-Plex can be used in its own right, whilst reducing the direct comparisons with MLST.

The methodology is sound and meets the expected standards. Enough detail is provided for the work to be reproduced.

Specific comments are listed below.

Authors compare the new Phylo-Plex scheme to the existing MLST scheme at multiple places of the manuscript. They conclude that the Phylo-Plex offers more resolution and is less costly. Data supporting these claims should be included.

Many thanks for this comment. As mentioned above we have now reduced the emphasis on comparing Phylo-Plex to MLST (Reviewer 2 question 2). We now highlight the phylogenetic consistency offered by this approach, as well as highlighting its value in reflecting global or local diversity underpinned by its resilience to missing data.

- It is unclear what the output of the Phylo-Plex typing is. Is it an assignment of an isolate to a TPA sub-lineage?

The output of the Phylo-Plex approach is a set of SNPs that are highly discriminatory between genomic clusters or sublineages which are positionally clustered to enable efficient recovery using multiplex PCR for the species this is applied to. These can be used for phylogenetic analysis or for explicitly defining a set of clusters (e.g. using a lookup table of SNPs). The method also allows the incorporation of virulence and AMR determinants, in addition to phylogenetically robust marker sequences. The latter relies on the question and the level of resolution required by the user when designing their Phylo-Plex amplicons using the system outlined in this paper.

-If this is the case, would it not provide lower resolution than MLST (17 TPA sub-lineages vs. over 110 different allelic profiles using MLST)? Clarification is needed.

This is an important question. As shown above, the MLST schemes designed for TPA suffer several flaws: they often rely on hypervariable genes that are frequently homoplasic with respect to the whole genome phylogeny (Lieberman et al, 2021). This is demonstrated above in the figure comparing Phylo-plex to MLST STs (please see figure in response to reviewer 1 question 2). Hence whilst MLST schemes find more individual STs than Phylo-Plex, they are not evolutionary or epidemiologically robust.

The reason for this difference is that for MLST you require 3-7 gene targets per bacterial species. When attempting to do this for TPA you have the added problem of it having a highly conserved genome with few genes showing enough variation on which to design a 3-7 gene MLST scheme. Hence, TPA MLST schemes have tended to select hypervariable or recombining genes because they contain the most variation.

The main innovation for Phylo-Plex is how the SNPs are combined to minimise the number of target amplicons built from the known diversity of the species and based on phylogenetically informative SNPs spread throughout the genome. Therefore, unlike MLST, Phylo-Plex does not rely on a small, set number of genes. It also explains why we are not advocating Phylo-Plex as a replacement for MLST: they perform different functions with both approaches having merit.

- Supplementary Figure 12: It would be helpful to construct the tree using the MLST scheme for comparison.

Many thanks. A tree built only from MLST alleles would, by definition, delineate groups but presenting such a tree could mislead readers to believe those clusters are phylogenetically robust, which they are not. We instead performed a comparison of MLST and Phylo-Plex sublineages against a WGS tree (see our earlier response above).

- A direct comparison of cited methods using the same set of clinical sample is warranted to highlight the better resolution of the Phylo-Plex when compared to the traditional typing methods.

As discussed above, we have now evaluated the 3-gene MLST scheme (Grillova et al) against a WGS phylogeny showing Phylo-Plex sublineages, but would prefer to exclude it within the manuscript because it would substantially change the focus of the paper without affecting the novelty and value of this approach.

- The lower price is highlighted by comparing Phylo-Plex via nanopore sequencing versus MLST via Sanger sequencing. These two sequencing methods have very different starting costs, which are unrelated to the typing schemes. What would be the cost if the MLST was performed using nanopore sequencing? If MLST uses 4 (3 discriminatory + 23S rRNA) instead of 59 amplicons, the overall price is expected to be lower.

Thank you for this important point. As mentioned earlier, Phylo-Plex is not intended as a replacement for MLST. However, it is correct that nanopore sequencing could also be applied to MLST. Phylo-Plex reagent costs (see Table 1; estimated at £12.15/sample for 24-sample Phylo-Plex) break down into the cost of the PCR reactions (£0.56/reaction, £13.32/24-samples), the cost of PCR primers (£0.18/reaction, £4.32/24-samples), the cost of library prep including indexing (£8.73/sample or £209.56/24-samples), and the cost of sequencing (£2.68/sample or £64.32/24-samples). In contrast to many methods where sequencing is the largest cost, here the largest cost comes from the library prep. We have now revised Table 1 to give a more detailed breakdown of Phylo-Plex costs.

For MLST, there are two scenarios in which nanopore sequencing might be applied. The most straightforward approach would be to adapt an existing 7-gene (or 3/4-gene in the case of Treponema) MLST – existing primers are unlikely to be compatible with multiplex PCR, and thus each gene would require discrete PCRs to be performed – seven PCRs instead of one – followed by pooling. The cost of performing seven PCR reactions would be £4.06/sample using our reagents. Alternatively, the primers for an existing MLST could be redesigned to enable a single multiplex-PCR reaction, in which case the PCR costs would be almost identical (reduced by £0.16/sample as fewer primers).

The other area to be impacted would that less sequencing depth would be required to cover 7 regions instead of 59. Since the price of a sequencing run on the MinION Flongle is fixed at £64.32 per run, costs savings could be made by increasing the number of samples per run. A 7-gene MLST could theoretically multiplex up to 192-plex on the MinION Flongle device, but this (i) assumes sufficient samples are available, and (ii) that sufficient indexes are available (currently ONT only provides a maximum of 96 indices).

We have now estimated costs for different scenarios (see table below and Supplementary Table 5 in manuscript). Although costings are very similar, applying an unaltered MLST (4- or 7-gene) to the same basic laboratory method for 24 samples would actually be more expensive (at £13.63/sample or £15.30/sample respectively) than the 59-amplicon TP-Phylo-Plex (£12.15/sample), and would remain so with a 7-gene MLST even for 192-plex, whilst a 4-gene

assay would need 96-plex to become cheaper per sample. Redesigning a 4-gene MLST to work as a single multiplex PCR would indeed be marginally cheaper than TP-Phylo-Plex, at £11.97/sample (24-plex) or £9.62/sample (192-plex).

Stage	Original PhyloPlex (single PCR, 24-plex)		4-gene_MLST (4 PCR rxns)				4-gene_MLST (multiplex PCR)			
	per 24	per sample	per 24	per 48	per 96	per 192	per 24	per 48	per 96	per 192
PCR (varies by PCR rxn and sample count)	£13.32	£0.56	£53.28	£106.56	£213.12	£426.24	£13.32	£26.64	£53.28	£106.56
PCR Primers (varies by PCR rxn and sample count)	£4.32	£0.18	£0.01	£0.02	£0.05	£0.10	£0.01	£0.02	£0.05	£0.10
Library (varies by sample count)	£209.56	£8.73	£209.56	£419.12	£838.24	£1,676.48	£209.56	£419.12	£838.24	£1,676.48
Sequencing (fixed)	£64.32	£2.68	£64.32	£64.32	£64.32	£64.32	£64.32	£64.32	£64.32	£64.32
Total (excluding PCR primers)	£291.52	£12.15	£327.17	£590.02	£1,115.73	£2,167.14	£287.21	£510.10	£955.89	£1,847.46
Cost per sample	**£12.15**	**£12.15**	**£13.63**	**£12.29**	**£11.62**	**£11.29**	**£11.97**	**£10.63**	**£9.96**	**£9.62**

Stage	Original PhyloPlex (single PCR, 24-plex)		7-gene_MLST (7 PCR rxns)				7-gene_MLST (multiplex PCR)			
	per 24	per sample	per 24	per 48	per 96	per 192	per 24	per 48	per 96	per 192
PCR (varies by PCR rxn and sample count)	£13.32	£0.56	£93.24	£186.48	£372.96	£745.92	£13.32	£26.64	£53.28	£106.56
PCR Primers (varies by PCR rxn and sample count)	£4.32	£0.18	£0.02	£0.04	£0.09	£0.17	£0.02	£0.04	£0.09	£0.17
Library (varies by sample count)	£209.56	£8.73	£209.56	£419.12	£838.24	£1,676.48	£209.56	£419.12	£838.24	£1,676.48
Sequencing (fixed)	£64.32	£2.68	£64.32	£64.32	£64.32	£64.32	£64.32	£64.32	£64.32	£64.32
Total (excluding PCR primers)	£291.52	£12.15	£367.14	£669.96	£1,275.61	£2,486.89	£287.22	£510.12	£955.93	£1,847.53
Cost per sample	**£12.15**	**£12.15**	**£15.30**	**£13.96**	**£13.29**	**£12.95**	**£11.97**	**£10.63**	**£9.96**	**£9.62**

Note that the cost of the TP-Phylo-Plex assay is £12.47 including plasticware, and £12.15 excluding plasticware – all costs in the table and discussed above are excluding plasticware (which is largely fixed).

In our discussion, we had already described how further changes to the library prep (e.g. replacing ONT indexes with in-house ones) might enable further cost reductions.

- The WGS price is listed for Illumina sequencing, which is again costlier than nanopore. What is the estimated price of sequencing TPA genome following DNA capture using nanopore?

Since sequencing prices vary widely, we quoted a generalised cost of sequencing based on an commercial provider (MicrobesNG, Birmingham, UK), who offer bacterial Illumina sequencing at £65/sample, and ONT sequencing at £85/sample (https://microbesng.com/whole-genome-sequencing/).

However, because TPA is a low abundance pathogen (typically <0.01% abundance) standard sequencing methods cannot be used without enrichment using bait capture. Approximately >98% of the ~2000 publicly available TPA genomes were sequenced this way. Whilst not offered commercially, the cost of using this method varies from £80-300/sample with Illumina and, to our knowledge, there are no published TPA genomes sequenced directly from clinical specimens using nanopore sequencing because of the limitations of enrichment with ONT.

The authors mention several times that their new approach can be applied to any pathogen. Treponema pallidum has very small and conserved genome with minimal differences, even between subspecies that cause distinct diseases. Authors state that more variable genomes would be even more suitable for Phylo-Plex development. However, the opposite is also possible with more Phylo-cons potentially needed to obtain the desired resolution, increasing price and data processing needs. It would be helpful to support this claim using a different pathogen. For example, estimate how many Phylo-cons would be needed to develop Phylo-Plex in a bacterium with more "standard" genome.

Thank you. We agree that *T. pallidum* has a very conserved genome, so to demonstrate this point we have now performed the *in silico* analysis portion of the Phylo-Plex workflow on a large dataset of the highly diverse and recombinogenic sexually transmitted bacteria, *Neisseria gonorrhoeae* (Tauok et al, 2024). In this study, the authors used cgMLST to define 1262 transmission clusters from a dataset of 5578 genomes. We selected all transmission clusters with ≥10 samples (n=79), and identified SNPs which defined these clusters, before using the Phylo-Plex approach to find information rich regions and select the optimal region set – for this highly diverse population we needed 169 Phy-cons to discriminate all sublineages. We have added a new section to the manuscript describing this replication (and the relevant methods) – see lines 349-371 and 660-681, and Supplementary figures 13, 14 and 15.

Finally, how are the results of Phylo-Plex evaluated in low-income settings?

As described in the paper, we evaluated the use of the method in a low resource laboratory in Zimbabwe, where all laboratory work and sequencing was performed, and where analysis was conducted on a laptop using a custom Nextflow pipeline with interactive 'Shiny' application for data interpretation.

Reviewer #3 (Remarks to the Author):

The authors developed a strategy to design a typing method based on targeted sequencing of phylogenetically informative regions across a set of genomes. Their method is focused on identifying target regions that are suitable for amplification in single-tube multiplex PCR reactions prior to sequencing. They use their strategy to design a typing scheme for T. pallidum, but their method could indeed be used for other organisms. Overall, I find the study scientifically sound and interesting. The authors follow a rational and phylogenetically-driven approach to build their typing scheme which, in my opinion, is one of the best ways a sub-genomic typing method could be developed (if not the best). I think the methodology of the authors overall makes sense, and their results overall back their claims. Therefore, I would recommend this work for publication after some corrections have been made to improve the manuscript, clarify some parts, and better discuss some points, in which I believe the authors may have not been completely fair. Importantly, I believe that the authors have missed relevant previous studies which should be discussed in their manuscript.

We thank you for your positive comments.

I hope that the authors will find our comments useful, all of which are constructively provided with the intention of improving the manuscript.

Sincerely,
Dr. Galo A. Goig and Gian Schüpbach

Major Comments:

The authors have missed relevant literature and should put their work in the context of previous studies (see Seabolt et al. Tau-typing: a Nextflow pipeline for finding the best phylogenetic markers in the genome for molecular typing of microbial species, Bioinformatics 2023).

Thank you for this – we added a note indicating the conceptual similarities to Tau-typing (lines 552-554).

The authors say they use a “globally representative dataset of T. Pallidum genomes”. The input dataset used for this kind of work is pivotal in the design of the scheme, however the authors do not provide references, nor explain, why the reader should believe that this dataset is globally representative. Also, representative of what exactly? Of the worldwide circulating genetic diversity? As for many other pathogens, T. Pallidum disproportionately affects LMICs, yet more than two thirds of the genomes analyzed in this study come from three high-income countries (Canada, The UK and USA). I understand that there may not be much WGS data available from LMICS, and I find acceptable that the authors just use what they have. But, is this dataset really representative of the global genetic diversity of T. Pallidum, given that for some high-incidence settings there are available no more than a couple of genomes? The authors should discuss the limitations of their typing scheme in this regard.

We agree with this point – our dataset was representative of all the available genomic data for TPA in 2022 (when we began work designing the scheme), but as we described in our 2021 manuscript (https://www.nature.com/articles/s41564-021-01000-z), global sampling from syphilis patients is heavily biased towards high income countries (HICs). Indeed, the overarching goal of our work was to improve representation of TPA genomes from LMICs, and we developed the Phylo-Plex framework as part of a surveillance project conducted in five African countries. Based on our previous findings (and those of more recent studies, e.g. Lieberman 2021, Taouk 2022, Beale 2023), it is fair to say that the dataset used is representative of TPA circulating in HICs, but is not representative for LMICs (e.g. it does not incorporate diversity sampled from LMICs in Lieberman 2021 or Sena 2024). We have now clarified this point when describing our dataset (lines 122-125).

I think the introduction of this paper lacks a couple of introductory sentences to i) the global genetic diversity of T. Pallidum. ii) The current typing methods available and more commonly used for T. Pallidum. This background is necessary to better evaluate the advantages of TP-Phylo-plex.

Done, we now include additional detail in the Introduction (lines 74-78).

Along the same lines, in the discussion section, the authors highlight the advantages of their methodology as opposed to other alternatives. I believe they are not fair in this comparison, by presenting overly pessimistic alternatives as compared to Phylo-Plex. For example, the authors say that MLST, “requires” sanger sequencing to then come up with an estimated cost of 28-112 pounds per sample, as compared to 12 of PhyloPlex. I do not see why MLST amplicons could not be sequenced with other technologies. As a matter of fact, the platform used by the authors (ONT) could be used for MLST schemes for the exact same reasons and with the same advantages and disadvantages than PhyloPlex. I believe the discussion should be rather focused on what value PhyloPlex adds as compared to alternatives such as MLST, but I doubt the decisive factor will be cost. Similarly, when the authors claim about Phylo-Plex: “because it can use multiple sequencing technologies, it can be scaled…” would apply to all other sequencing-based methods as well.

Thank you for this point. As we described in our response to Reviewer 2 above, ONT sequencing could indeed be applied to MLST schemes. From our calculations, unless the MLST primers are redesigned to work in multiplex PCR, the individual PCR reactions for a 3/4- or 7-gene MLST scheme would actually make the assay slightly more expensive than Phylo-Plex in most scenarios. However, we agree that cost is only one aspect of this work, since our method offers

far more potential resolution and phylogenetic consistency than can be achieved from sequencing 3- or 7-genes in MLST.

We also agree with the reviewer that cost is not the only consideration, nor is PhyloPlex necessarily a direct replacement for MLST. PhyloPlex is flexible and tunable to a particular population, whilst MLST is (by design) fixed and unchanging. This enables different use cases, with some overlap in the middle. We have now tried to emphasise this throughout the manuscript, including in the concluding sentence of our Discussion.

I do not understand the rationale to exclude the 15 amplicons from the initial Phylo-Plex scheme. In Supplementary Figure 9, it is very clear that the amplification efficiency for some amplicons is poor, and therefore the authors exclude them from the scheme. This is very clear for amplicons 1, 16, 37, 49, 67, 71, and maybe 38. However, some other amplicons do not seem problematic to me at all, and still the authors decided to exclude them in principle because they are part of the 5% that performed worst if I understood correctly. It does not seem to be a reasonable criterion to exclude regions, since even if all amplicons perform perfectly well, there will be always a 5% performing worst. For example, why were regions 20, 50 or 61 excluded? This is particularly relevant, given that exclusion of original amplicons led the authors to a rather disappointing discrimination power of around 75% of the original target.

Thank you for this.

Our apologies, we believe this misunderstanding comes from the legend for Supplementary Figure 9, which was incorrect. Hence, we agree the criteria for removal of 15 amplicons was inadequately described in our original manuscript, and we have now revised the Methods and Supplementary Figure legends to address this.

To identify poorly performing amplicons, we first identified and excluded four samples which performed poorly across all amplicons based on an overall mean coverage of <50X. By doing this, we ensured the analysis was not substantially biased by sample. Amongst the samples which performed well overall, we then identified poorly performing amplicons by asking what proportion of amplicons met a minimum coverage threshold of 25X (using the quantile function in R). We then excluded amplicons where >10% of the samples had a mean coverage below 25X. This is now described in the Methods on lines 831-839.

This is stringent and may have led to exclusion of some amplicons unnecessarily. At the time, we applied a very stringent cutoff to enable the laboratory validation to proceed ahead of the field test in Zimbabwe. This served well as a proof-of-principle, but in the long term a revised scheme for *T. pallidum* would be needed with further optimisation of poorly performing PCRs and additional genomic diversity which has become available since the scheme was originally designed. We now make clear in the text that this was for the purposes of scheme evaluation.

I also find confusing that the authors say in the text that, after exclusion, they discriminate 31/40 sublineages, but in Supplementary Figure 4 only 8, instead of 9, are pointed as removed.

Thank you for this point. It is important to recognise that although we focussed on selecting regions based on discriminatory SNPs with high F_{ST}, the regions we select also still contain other SNPs with lower F_{ST}, some of which may vary within or between sublineages, and this is part of the power of the method. In the manuscript, we inferred SNPs with very high discriminatory power in the dataset (i.e. $F_{ST} \geq 95$), and these are quantified for each sublineage using the different

SNP sets as shown in Supplementary Figure 4 (now labelled Supplementary Figure 5 in the revised manuscript). This approach shows that sublineages 1, 2, 3, 4, 6, 11, 14 and 35 had no remaining highly discriminatory SNPs in the final PCR scheme (i.e. 8 sublineages). In our later analysis (originally Supplementary Figure 11, now Supp Fig 12), we examined the complete sequence for the inferred amplicons (i.e. not just the highly discriminatory SNPs in our list) and came up with a different number. We failed to notice and investigate this, and are grateful to the reviewers for spotting this issue. We have now corrected this issue and have expanded the methods to clarify this point (for a more detail treatise of how this happened please see below).

To explain to you how this happened and why *T. pallidum serves as such a great exemplar for finding discriminatory SNPs used in this scheme:*

T. pallidum genomic data is by its very nature metagenomic, recovering extremely low abundance pathogens from mixed specimens. This is because very few isolates can be adapted to tissue culture. Even for genomes recovered with high sequencing coverage directly from culture, it is common for 'high quality genomes' to contain reference sites that remain undetermined, designated 'N' (we only included genomes with ≤5% of reference sites masked to 'N' in our analysis, although it is common to allow many more in other analyses). When we compared the SNP differences between the *in silico* inferred full-length amplicons, we used pairsnp (https://github.com/gtonkinhill/pairsnp, a fast implementation of the widely used snp-dists) to infer differences, and then created a network of identical distance clusters to track profiles. When pairwise comparisons contain 'N' sites in pairsnp/snp-dists they are not counted by default, and this can lead to misleading distances in data that contains a large number of 'N's. We overlooked this when performing our analysis, and this led to a small number of amplicon profiles being incorrectly labelled as identical/non-identical, with a knock-on effect on the profile uniqueness.

Since this is largely a result of the underlying dataset used for the *T. pallidum* design containing uncertain sites, to resolve this we have refined the variant calls at those positions by using joint ancestral reconstruction (https://github.com/simonrharris/pyjar) to infer the likely alleles at the uncertain sites for this particular analysis. This has enabled us to reanalyse the *in silico* generated amplicon profiles, and indicates that only four sublineages (sublineages 10 &11; 39 & 40) cannot be resolved. This now described in the methods (lines 692-695), and we have updated Supp Figure 12 and the relevant Results (lines 329-333). We are grateful to the reviewers for identifying this issue, and the updated analysis now suggests the performance *in vitro* would be better than previously stated.

I think that readers that do not realize that the strength of this work is its approach to design typing schemes, and not the scheme itself (which performs poorly as it is), may find the results disappointing: at the end of the day all this work to "only" discriminate 75% of the initial target sublineages. I would suggest to make this point very clear in the manuscript, and also discuss that a multiplex reaction always needs optimization (different concentrations of primers, inclusion of other amplicons from the initial pool of >800 amplicons, etc), something that was not carried out in this work. I only found such a comment in the legend of supplementary figure 4.

Thank you for this point – we have now strengthened this point on Lines 568-571.

Together with this low "sensitivity" of the scheme in its actual form, the authors report 3 samples

with discordant calls between Illumina and ONT. This means 5% of the samples tested. The authors say they implemented improvements in the pipeline to account for problematic samples with low coverage in the future. I am not sure if these improvements are the ones described in the Methods section but most importantly, instead of limiting to say that these improvements will deal with these problematic samples in the future, why did not the authors reanalyze the data and demonstrate that these improvements are able to deal with these discrepancies in this present study?

This is a good point, and we have now repeated the analysis using the updated pipeline. However, the reason for one of the discrepant sites (916096) was insufficient coverage in the ONT sequencing – rerunning the pipeline does not resolve this issue (nor would we want it to give unsupported SNP calls).

The reimplemented nextflow pipeline ensures that the consensus sequence generated for these low coverage sites are masked to 'N'. With respect to the other discrepancy described (7002), this was a deletion that was not detected by the ONT pipeline. Since Phylo-Plex analysis focusses on SNPs, the value of INDELS in this setting is questionable, and the revised pipeline does not resolve this. We have revised the text on lines 298-307 to make this clearer.

"The median genome count for each window was 129 (reflecting the number of T. pallidum genomes in the dataset), and regions of the T. pallidum 23S operon identified as matching ≥5% above the median (135.5) were flagged as potentially cross-species matches and masked to avoid use in primer design" If the number of TP genomes in the dataset is 129, why is 129 not the threshold, instead of 5% of the median? That would mean that around 6 species which are not TP may amplify, and precisely given how conserved this gene is, and its implications in drug resistance, I do not think this is an appropriate criterion.

This refers to the identification of regions of the 23S ribosomal operon that were specific to *T. pallidum*, and we apologise for the lack of clarity here. The 661K dataset published by Blackwell and colleagues contains 174 assemblies classified as *Treponema* of which 125 were classified as *T. pallidum*. We did not consistently observe hits for all 125 *T. pallidum* genomes (likely related to the *Tp* assemblies in the dataset being based on metagenomic data), and the median of 129 identical (100% identity) hits observed per window represents hits both within and between *Treponema* species.

Our goal here was to exclude regions that included non-*Treponema* hits, since clinical metagenomic samples from genital swabs are highly likely to contain other genera, whilst being less likely to contain non-pallidum *Treponema*. Indeed, one window resulted in 229,791 identical hits, of which 51,460 were to *S. pneumoniae* and 48,124 were from *S. aureus*. Therefore, our primary concern was to exclude windows with excessive hits outside the *Treponema* genus. We used the crude metric of 5% ≥ the median as an initial screen for excluding windows, and with hindsight, we could have applied a more specific initial screen to exclude windows on the basis of non-Treponema hits. Importantly, this was only an initial screen, and we also examined the species/genus hits recovered, as well as performing a thorough screen of final primers using primer BLAST. This enabled us to evaluate the likely impact of those hits (e.g. the likely ecological niche) and further exclude regions. We have now clarified further the reasoning and that the primary check for specificity used here was taxonomic classification and primer BLAST, not the raw COBS hit count (lines 646-659).

Minor comments:

The authors say that the TP-Phylo-Plex scheme was straightforward for TP given the low genetic diversity and little recombination. They could comment on how feasible would be to design schemes for pathogens with higher diversity.

We agree, and also in response to Reviewer 2, we have now applied the same *in silico* approach to a highly diverse *Neisseria gonorrhoeae* transmission study, demonstrating the generalisability of the method. Notably, applying the computational code to such a large (5,880 genomes) and diverse (88,671 variant sites) dataset was considerably more computationally demanding than for *T. pallidum*, and some of the code has been rewritten for more optimal use on a computer cluster. This analysis is now described in the Results (lines 349-371), as well as in the Methods (lines 660-681).

"Notably, those sublineages occurred in clonal lineages such as TPA SS14-Lineage which had the smallest number of available discriminatory sites even at the WGS level, and were thus most affected." I find this statement misleading. In the tree of Supplementary Figure seems that most of the unclassified samples would have enough diversity and therefore that the reason why discrimination was not possible was rather the exclusion of the amplicons that contained their defining SNPs, no the fact that discrimination is impossible between highly clonal strains.

In Supplementary Figure 5 we show the number of discriminatory SNPs available for each sublineage at each stage. For example, Sublineage 1 and 2 each had only one discriminatory SNP even before region selection, and this reflects extremely short branch lengths within this part of the tree. Whilst removal of amplicons does indeed explain why the sublineages are no longer discriminated, those sublineages most affected are the ones that already had limited discrimination because of low diversity. We have now clarified this sentence on lines 329-339.

"Then, regardless of the nature of any individual SNP or gene, all discriminatory SNPs are positionally clustered..." What do the authors mean by "the nature of the SNPs" exactly? This is unclear, and if they mean "regardless of the genomic region in which these SNPs are", then it would not be accurate since their analysis explicitly excludes hypervariable, recombining and repetitive regions.

This statement was intended to convey that we were considering all discriminatory SNPs as a population, rather than by what sublineage they supported (or indeed what region they belonged to). We have now changed this sentence for clarity on lines 100-101.

The authors built a ML phylogeny with IQ-TREE, but it is not specified the model of evolution, bootstrap replicates, etc...

Done, please see revised methods lines 596-598.

"To identify highly conserved Treponema specific regions in the 23S gene, which occurs universally at high homology across bacteria" please substitute homology by another term such as similarity. A trait is either homologous or non-homologous, it cannot be more or less homologous.

Done.

I suggest to improve figure 8 by overlaying the points to boxplots. I also suggest to include a plot where the correlation (or lack thereof) between qPCR Ct and average sample depth is shown. Supplementary 8, together with Suppl 9, show no correlation between Ct and depth, this means that Supplementary Figure 9 could be simplified and would be more informative if eliminating the X axis (Ct), and simply plotting boxplots and datapoints of depth for each amplicon.

Thank you for these suggestions. We originally used boxplots for displaying these data, but found that they did not adequately show the distribution of the data and made the plots overly crowded. Notably, Supp Figure 9 (previously 8) contains data from three separate ONT sequencing runs (corresponding to three groups of samples with high, medium and low qPCR Ct), with the samples ordered by both qPCR Ct and also by run. To address this point, we have made the distinction between different sequencing runs much clearer in the plot through faceting, as well as adding a median coverage marker line for each sample and removing sample names (since these were not informative).

Although we agree that there is no clear correlation across all amplicons (Supplementary Figure 9), we believe that Supplementary Figure 10 demonstrates a non-linear relationship between qPCR and coverage for individual amplicons, with high Ct samples giving reduced sequencing performance. We have therefore retained Supplementary Figure 9 in its original form.

In the bibliography, species names are not in italics.

Done.

Reviewer #4 (Remarks to the Author):

I co-reviewed this manuscript with one of the reviewers who provided the listed reports. This is part of the Nature Communications initiative to facilitate training in peer review and to provide appropriate recognition for Early Career Researchers who co-review manuscripts.

Reviewer #5 (Remarks to the Author):

The manuscript entitled "Phylo-Plex: A phylogenetically informed, low-cost amplicon sequencing platform for deployable high-resolution genomic epidemiology" describes a framework for the design of an amplicon panel for targeted culture-independent sequencing. This framework is focused on the acquisition of genomic information though the identification of ancestral SNPs, that will be useful for the genomic epidemiology of pathogens of public and global health concerns. The authors applied this framework to develop a multiplex PCR panel capable of distinguishing between sub lineages of T. pallidum. This resulted in a final pool of 59 primer set. The authors further validated the panel in a low-resource laboratory setting, demonstrating its ability to recover amplicon profiles from clinical samples with Ct values ≤32, consistent with their initial validation.

The manuscript reads well and the framework will be a very useful tool for the design of amplicon panels, targeting hard-to-culture pathogens (with good genomic representation in public genomic repositories). While I recommend publication, there are a few (mostly minor) comments to be addressed.

Thank you for your positive summary.

Major comments

• Dropping the number of amplicons from 72 resulted in the loss of discrimination from 9 of the initial targeted 40 sub lineages. As the authors mentioned that singleplex PCR all performed ok, was the generation of multiple primer pools to reduce primer cross interactions considered/investigated to mitigate this loss in resolution?

Yes, we considered both further optimisation of the multiplex PCR and also the addition of further multiplex pools (which would of course increase the cost of the assay). For the purposes of the field evaluation, we eliminated the poorly performing primer pairs, but a long-term goal would be to revise the scheme to reincorporate some of those regions as well as include additional genomic diversity that has been described since the scheme was designed.

• Targeted culture-independent sequencing can also be achieved through other methods via ONT sequencing. One such method is ONT Adaptive Sampling, which requires no prior primer amplification and uses the same hardware for sequencing. While the role of amplicon sequencing for public health purposes is not in doubt, deeper discussion points between the various culture independent sequencing methods would provide valuable context and enhance the depth of the discussion.

We agree that there are alternative approaches available for enriching sequencing data from mixed specimens. Büttner et al. (https://www.microbiologyresearch.org/content/journal/mgen/10.1099/mgen.0.001353) recently compared metagenomics, bait capture and ONT Adaptive Sequencing for STI pathogens (including *Chlamydia* and *T. pallidum*). In the case of low abundance pathogens such as *T. pallidum* (typical abundance of <0.01%) ONT Adaptive Sequencing does not provide sufficient enrichment to make this affordable. *T. pallidum* genomes are typically sequenced using bait capture (enabling 100-1000-fold enrichment), which costs £80-300 per genome, and this would also not be affordable for a low resource setting. Given the recent comparison, we have added discussion of alternatives in our Discussion, including citing Büttner et al and others (Lines 509-515).

Other comments

• Page 7: It was mentioned in-text that there were 72 amplicons and 1x 23S amplicon. However, supplementary table 2 had 72 amplicons and 2 x 23S amplicons listed. The second 23S amplicon did not make it into the final set and was not mentioned in-text. Why was this primer dropped?

Thank you for spotting this omission. The 23S primers were designed separately to the rest of the primers using the same settings and we were concerned about compatibility, since the version of PrimalScheme used does not allow consideration of existing primers when designing new primer sets. We therefore designed two different versions of 23s amplicon, which we separately evaluated as part of the multiplex. We ultimately selected the 23S amplicon which performed best. We have now clarified this in our Methods on lines 657-659.

• Page 10: This sentence needs clarification: "Of the two discrepant sites, the first (NC_021508.1 position 7002) affected 1/59 samples and was due to a deletion detected in the Illumina sequence (7002T) compared to the reference and MinION sequences (7002TG)." Do the authors mean that a T deletion at position 7002 (7002delT) was observed only in the Illumina reads and not in the ONT reads (prior to closer inspection)? If so, I recommend referring to it as 7002delT

and omitting the “7002TG” notation, since positions 7002 and 7003 in NC_021508.1 are T and G, respectively.

Thank you for this suggestion – we used this description because of the way the Clair3 variant caller described the variant. For clarity, it was 7003G in the MinION data and Reference, and 7003delG in the Illumina data – we have now clarified this on lines 292-307.

• Page 13: During the implementation study, two samples had one technical replicate each and one sample had three replicates. Two samples (mz454 & mz446) had CT values that are different within their replicates (from Supplementary Table 4), are these values listed in supplementary table correct or are they biological replicates? In addition, why did three samples have replicates but not the others?

These samples are different types of technical replicates – either independent extractions of the same swab, or independent tests of the same extraction, and the discrete extractions have minor variation in qPCR Ct, as would be expected. Samples 454a and 454b are technical replicates from different extractions (F4 and A8) of the same sample. Samples mz446a and mz446c are technical replicate tests from the same extraction (F13), whilst sample mz446b is a technical replicate of the same sample but a different extraction (E5). Samples mz476a and mz476b are technical replicates from the same extraction (D4).

For each patient, we had two aliquots of frozen swab lysate (derived from the same original swab) for 100 samples. After DNA extraction of the first swab, we quantified the total DNA using Nanodrop – samples with very low total DNA were reextracted using the second swab in case this performed better. During the implementation study, we identified 14 *T. pallidum* positive samples, but after performing repeat extractions and qPCRs we were constrained for time in Zimbabwe, and able to perform a single *T. pallidum* sequencing run.

Since our sequencing method was developed assuming 24-samples per run, we had room to include up to 10 replicates, but we focussed on replicate samples we had available, ultimately preparing 18 libraries. Our rationale was to select a mix of replicates for samples with both high and low total DNA and Treponema qPCR load, and we selected two samples with low load and one with high load – we were also able to include a mix of different types of technical replicates. We apologise for not making this clearer, and have now clarified this on lines 402-408 in the Results, on lines 719-724 in the Methods and in the Supplementary Table 4.

• Page 13: From cross checking of the data in Supplementary Table S4, did the authors mean “5 samples, 3 of which are replicates of a single sample” instead of “(3 samples (5 replicates)” In addition, mz454a with a CT of 25 (PolA) also resulted in a failed run, which should be mentioned in text.

Thank you – we have now clarified this in the Results on lines 402-408.

• Page 13: Authors mentioned that 12 TP-Phylo-Plex profiles were generated without going into details on the sublineages called. Can the authors report on the sub lineage calls of each of the profile (either in text or in supplementary).

The samples were from Zimbabwe, and represented novel sublineages compared to those used during the design of the TP-Phylo-Plex scheme. Given that they were novel, our analysis

represented them as two distinct phylogenetic clades distinct from the diversity known at the time of scheme design. We have added a note to describe this point on lines 412-413.

Also, one of the samples that was successful in sequencing also had a technical replicate- can the authors comment if the technical replicates resulted in the same sublineage call?

The technical replicates that successfully sequenced (sample mz476) gave identical SNP profiles in the amplicons. This detail has been added to the results.

• Page 13: The estimated cost per sample does not include DNA extraction. It is understandable why this was not included as the focus of the implementation was on primer scheme. I recommend that the following be added to the sentence for completeness "...(Table 1), in addition to existing costs for DNA extraction and quality control."

Done.

• Page 21: DNA extraction method not listed within laboratory workflows. Please include details on extraction methodology used in this study.

We used QIAamp spin columns - this has now been added to lines 726-730.

• Page 22: Change the catalogue numbers of the Native barcoding library preparation kits to the SQK designations instead of the auxiliary EXP designation.

Done.

• Page 23: Please include the model used in Clair3 for the processing of the R9 data.

Done.

• Supp. Figure 8: In text, the quality metric mentioned was <50X but the legend for the supplementary figure stated that a blue line was drawn at 25 X. Do provide an updated figure with the line drawn at 50X to be in-line with what was mentioned in-text.

Done.

• Supp. Figure 12: Image resolution of the minimum spanning tree in Panel B is lower than Panel A. Please provide a higher resolution figure for Panel B.

This reflects the way R has plotted vector graphics generated externally as a combined figure. We have tried to improve this by changing to linear interpolation, and will provide individual .svg files as requested during production.
